# Molecular validation of clinical *Pantoea* isolates identified by MALDI-TOF

**DOI:** 10.1371/journal.pone.0224731

**Published:** 2019-11-04

**Authors:** Craig D. Soutar, John Stavrinides

**Affiliations:** Department of Biology, University of Regina, Regina, Saskatchewan, Canada; Universidade de Coimbra, PORTUGAL

## Abstract

The Enterobacterial genus *Pantoea* contains both free-living and host-associating species, with considerable debate as to whether documented reports of human infections by members of this species group are accurate. MALDI-TOF-based identification methods are commonly used in clinical laboratories as a rapid means of identification, but its reliability for identification of *Pantoea* species is unclear. In this study, we carried out *cpn60*-based molecular typing of 54 clinical isolates that had been identified as *Pantoea* using MALDI-TOF and other clinical typing methods. We found that 24% had been misidentified, and were actually strains of *Citrobacter*, *Enterobacter*, *Kosakonia*, *Klebsiella*, *Pseudocitrobacter*, members of the newly described *Erwinia gerundensis*, and even several unclassified members of the *Enterobacteriaceae*. The 40 clinical strains that were confirmed to be *Pantoea* were identified as *Pantoea agglomerans*, *Pantoea allii*, *Pantoea dispersa*, *Pantoea eucalypti*, and *Pantoea septica* as well as the proposed species group, *Pantoea latae*. Some species groups considered largely environmental or plant-associated, such as *P*. *allii* and *P*. *eucalypti* were also among clinical specimens. Our results indicate that MALDI-TOF-based identification methods may misidentify strains of the *Enterobacteriaceae* as *Pantoea*.

## Introduction

Strains of *Pantoea* are frequently found in association with a wide variety of hosts, including plants, insects, animals, and humans [1,2]. Multiple *Pantoea* species are well-documented plant pathogens [3–5]; however, *Pantoea* species have also been isolated from clinical specimens. *Pantoea agglomerans* has been isolated from pediatric patients with bacteremia, osteomyelitis, peritonitis, pneumonia, septic arthritis, and septicemia [6]. The majority of *P*. *agglomerans* clinical cases are either the result of wound contamination with plant material or are hospital-acquired infections [7]. Likewise, other species such as *Pantoea ananatis*, *Pantoea dispersa*, *Pantoea eucalypti*, and *Pantoea septica* have also been isolated from a variety of clinical sources including wounds, blood and other fluids, skin, stool, abscesses, cysts, fractures and body sites such as the urethra and trachea [2]. *Pantoea* has also been implicated in multiple outbreaks that resulted in the deaths of neonates [8,9]. Despite this, the human pathogenic potential of many *Pantoea* species is currently being debated as there is evidence to suggest that many clinical strains are not *Pantoea* at all [10]. Due in part to taxonomic and nomenclatural revisions many strains previously listed as *Pantoea* have been found to belong to other genera including *Klebsiella* and *Enterobacter* [11]. Furthermore, *Pantoea* strains are difficult to assign to a species group based on metabolic profiling alone, which has resulted in numerous *Pantoea* clinical isolates being incorrectly classified as *Pantoea agglomerans* [12].

Identification of clinical specimens of *Pantoea* is often achieved by the mass spectrometry-based approach matrix-assisted laser desorption ionization-time of flight (MALDI-TOF). MALDI-TOF involves the application of a laser to an isolated bacterial colony that has been treated with a matrix solution, leading to ionization of bacterial molecules that are then used as the signature for genus- and/or species-level identification via comparison to a reference database [13,14]. MALDI-TOF can achieve species-level identification with greater accuracy and speed than conventional biochemical methods [15,16]. However, as MALDI-TOF identification relies upon a database of profiles of known reference organisms, gaps in the database can lead to misidentification. Indeed, many reported MALDI-TOF misidentifications are the result of incomplete databases with most situations remedied by updating the reference database with additional organisms [15,17]. Likewise, novel and undescribed genera and species may not be discernible from the next closest relative. For the genus *Pantoea*, which continues to be revised with new species groups, the accuracy of MALDI-TOF-based identifications remains unknown.

Molecular typing methods can be used to help validate the accuracy of MALDI-TOF based clinical identifications of *Pantoea*. The 16S ribosomal RNA (rRNA) gene is considered to be a universal identifier for bacteria largely due to its conservation across Bacteria, and due to the phylogenetic signal provided by the approximately 1500 base pair (bp) locus [18]; however, this slowly-evolving locus often does not provide sufficient resolution for distinguishing between *Pantoea* species groups [19,20]. In contrast, the multi-locus sequence analysis (MLSA) approach has demonstrated reproducible typing of strains along with robust phylogenies for *Pantoea* [21]. Single-gene barcoding of *Pantoea* using *leuS* has also been proposed, which provides consistent species identification with some incongruencies only in the relative position of particular species groups in the tree [22]. The *leuS* gene, however, has not been developed as a universal marker for bacterial identification and classification, making comparisons across species and across studies considerably more difficult with this locus. In contrast, *cpn60*, known as *groEL* in *E*. *coli*, is a roughly 1650 bp chaperonin gene that has been shown to reliably provide robust species-level resolving power [23–25]. This gene is present in the genome of almost all bacteria and contains a region of close to 600 bp that has been designated a universal target for discriminating between closely related species [26]. In addition, a curated database of *cpn60* sequences is available online [27].

In this study, we performed species-level identification of clinical and environmental candidate *Pantoea* isolates using a combination of MLSA and *cpn60*-based typing. We first generated and compared a *cpn60* gene genealogy of *Pantoea* reference strains to a phylogeny generated by MLSA to demonstrate that *cpn60* consistently recovers the *Pantoea* species groups with strong support. We used this robust clustering of species groups to type 64 candidate *Pantoea* strains from clinical and environmental sources, and show that 24% of clinical isolates were misidentified, with MALDI-TOF misidentifying one of every five strains. Of those strains that were correctly identified, the majority were *P*. *agglomerans* and *P*. *septica*. We also found clinical strains of the plant-associated, *Pantoea allii* and *P*. *eucalypti*.

## Materials and methods

### Bacterial strains

Clinical isolates were obtained from St. Boniface General Hospital in Winnipeg, Manitoba, Canada, the Texas Children's Hospital in Houston, Texas, USA, the Roy Romanow Provincial Laboratory in Regina, Saskatchewan, Canada, and the Regina General Hospital in Regina. Strain information provided included a tentative identification to genus, as well as anonymized patient information (Table 1). Clinical identification of isolates by St. Boniface General Hospital was achieved via a Bruker Biotyper microflex LT/SH MALDI-TOF system, which used the RUO MALDI Biotyper Reference Library (Bruker Ltd., Milton, Ontario, Canada). Texas Children's Hospital identified isolates using a combination of VITEK 2 [28] and 16S rRNA gene sequencing, the Roy Romanow Provincial Laboratory with MicroScan [29], biochemical typing, and 16S rRNA gene sequencing, and the Regina General Hospital with VITEK 2 ID cards. Environmental strains that were initially identified as *Pantoea* via phenotype-based methods were obtained from a variety of sources. Reference strain genomes were obtained from NCBI and our lab collection (S1 Table).

**Table 1:** Candidate *Pantoea* isolates used in this study.

Strain	^a^E/C	^b^Probable species	^c^Host/Locale	Source	^d^Initial ID method
07–703	C	unclassified *Enterobacteriaceae*	Human, 82 years old, blood, Abdominal pain (non-trauma)	St. Boniface General Hospital, Winnipeg, MB, Canada	MT
10–854	C	*Pseudocitrobacter*	Human, 58 years old, abdominal fluid, Ruptured appendix	St. Boniface General Hospital, Winnipeg, MB, Canada	MT
12BT205805	C	unclassified *Enterobacteriaceae*	Human, 62 years old, ETC, Arrest, Hypothermia	St. Boniface General Hospital, Winnipeg, MB, Canada	MT
12DB793227A	C	*Pantoea agglomerans*	Human, 68 years old, ETC, MI	St. Boniface General Hospital, Winnipeg, MB, Canada	MT
12DB793227B	C	*Pantoea agglomerans*	Human, 68 years old, ETC, MI	St. Boniface General Hospital, Winnipeg, MB, Canada	MT
12GB497105	C	*Kosakonia*	Human, 54 years old, fluid, Gallbladder	St. Boniface General Hospital, Winnipeg, MB, Canada	MT
12GC134883	C	*Citrobacter*	Human, 98 years old, Edema, CHF, Cellulitis	St. Boniface General Hospital, Winnipeg, MB, Canada	MT
12VB493046	C	*Pantoea dispersa*	Human, 54 years old, ETC, Post PCI, Cardiac Arrest	St. Boniface General Hospital, Winnipeg, MB, Canada	MT
12VB899147A	C	*Pantoea septica*	Human, 82 years old, urine	St. Boniface General Hospital, Winnipeg, MB, Canada	MT
13BC160225A	C	*Pantoea septica*	Human, 59 years old, urine, Febrile neutropenia	St. Boniface General Hospital, Winnipeg, MB, Canada	MT
13BG284532	C	*Pantoea* sp.	Human, infant, trach secretion, Premature	St. Boniface General Hospital, Winnipeg, MB, Canada	MT
13DB433109	C	*Pantoea* sp.	Human, 69 years old, urine midstream, Flank pain	St. Boniface General Hospital, Winnipeg, MB, Canada	MT
13DB759184B	C	*Pantoea septica*	Human, 74 years old, nephrostomy urine, Renal failure	St. Boniface General Hospital, Winnipeg, MB, Canada	MT
13DB759184C	C	*Pantoea septica*	Human, 74 years old, nephrostomy urine, Renal failure	St. Boniface General Hospital, Winnipeg, MB, Canada	MT
13DB767309C	C	*Pantoea septica*	Human, 74 years old, blood culture, Renal failure	St. Boniface General Hospital, Winnipeg, MB, Canada	MT
13VB752675	C	*Pantoea agglomerans*	Human, 60 years old, coccyx wound, Sepsis	St. Boniface General Hospital, Winnipeg, MB, Canada	MT
14BB43300	C	*Pantoea septica*	Human, 54 years old, sputum	St. Boniface General Hospital, Winnipeg, MB, Canada	MT
14GC287951	C	*Pantoea septica*	Human, 92 years old, urine midstream, Fall	St. Boniface General Hospital, Winnipeg, MB, Canada	MT
14MB215572	C	*Erwinia gerundensis*	Human, 27 years old, eyes, Peri-orbital swelling	St. Boniface General Hospital, Winnipeg, MB, Canada	MT
14VB542579A	C	*Klebsiella*	Human, 62 years old, catheter urine, Cystocopy stent insertion	St. Boniface General Hospital, Winnipeg, MB, Canada	MT
14VB542579B	C	*Bacillus*	Human, 62 years old, catheter urine, Cystocopy stent insertion	St. Boniface General Hospital, Winnipeg, MB, Canada	MT
15BB477046	C	*Mixta calida*	Human, 26 years old, urine midstream, Abdominal pain	St. Boniface General Hospital, Winnipeg, MB, Canada	MT
15DB693365	C	*Pantoea eucalypti*	Human, 37 years old, bronch wash, Cough	St. Boniface General Hospital, Winnipeg, MB, Canada	MT
15IE404477	C	*Pantoea septica*	Human, 54 years old, leg ulcer, Localized swelling	St. Boniface General Hospital, Winnipeg, MB, Canada	MT
15MB93116	C	*Pantoea septica*	Human, 56 years old, foot ulcer to bone	St. Boniface General Hospital, Winnipeg, MB, Canada	MT
15VB680695	C	*Pantoea agglomerans*	Human, 81 years old, arm wound, Hemochromatosis	St. Boniface General Hospital, Winnipeg, MB, Canada	MT
16BD751234	C	*Pantoea allii*	Human, 56 years old, maxillary sinus, Chronic sinusitis	St. Boniface General Hospital, Winnipeg, MB, Canada	MT
16BF887461	C	*Pantoea* sp.	Human, 1 year old, blood culture	St. Boniface General Hospital, Winnipeg, MB, Canada	MT
16DB688514	C	*Pantoea agglomerans*	Human, 47 years old, ortho knee, infected	St. Boniface General Hospital, Winnipeg, MB, Canada	MT
16GB300243	C	*Pantoea septica*	Human, 80 years old, penis	St. Boniface General Hospital, Winnipeg, MB, Canada	MT
16GB303624	C	*Pantoea septica*	Human, 80 years old, midstream urine	St. Boniface General Hospital, Winnipeg, MB, Canada	MT
16GB504195	C	*Pantoea septica*	Human, 70 years old, catheter urine	St. Boniface General Hospital, Winnipeg, MB, Canada	MT
16IE448486	C	*Pantoea septica*	Human, 43 years old, incision wound, Hysterectomy	St. Boniface General Hospital, Winnipeg, MB, Canada	MT
16MB259464	C	*Pantoea septica*	Human, 35 years old, nostril wound	St. Boniface General Hospital, Winnipeg, MB, Canada	MT
16MB264552	C	*Pantoea agglomerans*	Human, 37 years old, wound—face sore	St. Boniface General Hospital, Winnipeg, MB, Canada	MT
17BE3618	C	*Pantoea septica*	Human, 71 years old, endotrach. secretions, Respiratory failure	St. Boniface General Hospital, Winnipeg, MB, Canada	MT
17BG299761-2	C	*Pantoea agglomerans*	Human, 74 years old, fistula, End-stage renal disease	St. Boniface General Hospital, Winnipeg, MB, Canada	MT
17DB268518	C	*Pantoea agglomerans*	Human, 61 years old, toe wound	St. Boniface General Hospital, Winnipeg, MB, Canada	MT
17DB651035	C	*Pantoea* sp.	Human, 62 years old, endotrach secretions	St. Boniface General Hospital, Winnipeg, MB, Canada	MT
17IE403177	C	*Pantoea* sp.	Human, 51 years old, midstream urine, Urinary tract infection	St. Boniface General Hospital, Winnipeg, MB, Canada	MT
17IE565985	C	*Pantoea septica*	Human, 34 years old, elbow	St. Boniface General Hospital, Winnipeg, MB, Canada	MT
17IE656463	C	*Pantoea septica*	Human, 1 year old, eye, Conjunctivitis	St. Boniface General Hospital, Winnipeg, MB, Canada	MT
17IE68873	C	*Pantoea septica*	Human, 9 years old, abdominal wall	St. Boniface General Hospital, Winnipeg, MB, Canada	MT
17MB171522-1	C	*Pantoea agglomerans*	Human, 51 years old, right foot wound	St. Boniface General Hospital, Winnipeg, MB, Canada	MT
17MB171522-2	C	*Erwinia gerundensis*	Human, 51 years old, right foot wound	St. Boniface General Hospital, Winnipeg, MB, Canada	MT
17VB530109	C	*Pantoea septica*	Human, 74 years old, catheter urine	St. Boniface General Hospital, Winnipeg, MB, Canada	MT
17VB556491	C	*Pantoea agglomerans*	Human, 66 years old, leg wound	St. Boniface General Hospital, Winnipeg, MB, Canada	MT
20S	E	*Pantoea agglomerans*	bumblebee	Regina, SK	P
22	E	*Pantoea agglomerans*	thistle	Regina, SK	P
23	E	*Pantoea agglomerans*	soil	Regina, SK	P
B011499	C	*Klebsiella*	Human female, 96 years old, urine indwelling catheter, Cloudy urine	Roy Romanow Provincial Lab, Regina, SK	MS+16S+BC
B012497	C	unclassified *Enterobacteriaceae*	Human female, 57 years old, urine midstream	Roy Romanow Provincial Lab, Regina, SK	MS+16S+BC
B23I	E	*Pantoea agglomerans*	soil	Regina, SK	P
B6I	E	*Acinetobacter*	algae from rock	Regina, SK	P
FB1	E	*Pantoea agglomerans*	flea beetle	Regina, SK	P
FB2	E	*Pantoea agglomerans*	flea beetle	Regina, SK	P
FB4	E	*Paenibacillus*	flea beetle	Regina, SK	P
FB6	E	*Pantoea agglomerans*	flea beetle	Regina, SK	P
G4061350	C	*Klebsiella*	Human—urine	Regina General Hospital, Regina, SK	V
ICMP12202	E	*Kosakonia*	Muskmelon (*Cucumis melo* L.)	ICMP, New Zealand	P
TX2	C	*Enterobacter*	Human—sputum (cystic fibrosis)	Texas Children's Hospital, Houston, Texas, USA	V+16S
TX7	C	*Pantoea latae*	Human—blood	Texas Children's Hospital, Houston, Texas, USA	V+16S
TX9	C	*Pantoea* sp.	Human—foot	Texas Children's Hospital, Houston, Texas, USA	V+16S
TX11	C	*Pantoea latae*	Human—sputum	Texas Children's Hospital, Houston, Texas, USA	V+16S

^a^ E = environmental; C = clinical.

^b^ Identification based on consensus of *cpn60* phylogeny, cpnDB best hit, and 16S rRNA RDP Classifier output.

^c^ ETC = Esophageal Tracheal Combitube; MI = Myocardial Infarction; PCI = Percutaneous Coronary Intervention; CHF = Congestive Heart Failure.

^d^ MT = MALDI-TOF; P = Pigmentation; MS = MicroScan; 16S = 16S rRNA gene; BC = Biochemical assays; V = VITEK 2

### Sequence data

The gene sequences of *atpD*, *fusA*, *gyrB*, *leuS*, *recA*, *rplB*, and *rpoB* as well as *cpn60* were extracted from *Pantoea* genomes from the National Centre for Biotechnology Information (NCBI) and from our collection [2] using an in-house Perl-based pipeline. Complete genomic data were not available for representatives of *Pantoea beijingensis* and *coffeiphila* so these were not included in the analysis. For new strains, the 16S rRNA and *cpn60* genes were amplified using primers 16S+335 (ACTCCTACGGGAGGCAGC) and 16S-1400 (ACGGGCGGTGTGTACAA) in a colony PCR reaction with New England Biolabs Taq DNA polymerase (New England Biolabs Ltd., Whitby, Ontario, Canada) as per the manufacturer’s instructions, and cpn60_ent+1 (ATGGCAGCWAAAGACGTAAAATTCGG) and cpn60-1330 (CGCRACYTTRATACCSACGTTCTG) in a colony PCR reaction with GenedireX Taq DNA polymerase (GenedireX Inc., Taiwan) as per the manufacturer’s instructions. Amplicons were sequenced using Sanger sequencing by Genome Quebec (Montreal, Quebec, Canada). Forward and reverse reads were merged using the BBMap software package [30]. MLSA loci and *cpn60* gene sequences have been deposited in Genbank under accession numbers MK909837-MK909900, MK928255-MK928322, and MK936803-MK936866.

### Sequence analysis and phylogenetics

16S rRNA gene sequences were analyzed using the Ribosome Database Project (Training Set 16) Classifier [31]. *cpn60* sequences were analyzed with a custom cpnDB database of Group I sequences [27] that included the *cpn60* sequence of *P*. *septica* strains FF5, VB38951-A, and X44686, as well as *Pantoea* sp. PSNIH6, *Pantoea* sp. RIT388, *Pantoea* sp. UBA4389, *Enterobacteriaceae* bacterium IIIF5SW, *Erwiniaceae* bacterium IIIF1SW-P2, *Izhakiella australiensis* D4N98, *Tatumella saanichensis* NML 06–3099, *Mixta calida* LMG 25383 *Pseudocitrobacter* sp. RIT 415, *Pseudocitrobacter faecalis* DSM 27453, *Erwinia gerundensis* EM595, and *Kosakonia cowanii* Esp_Z (S1 Table). Alignments for phylogenies were generated using Clustal Omega version 1.2.1 using default parameters [32]. Alignments for the MLSA trees consisted of concatenated full-length *fusA*, *gyrB*, *leuS*, *recA*, *rplB*, *rpoB*, and *atpD* gene sequences, while *cpn60* alignments contained sequences of at least 530 bp of the coding sequence. Maximum likelihood trees were constructed in MEGA version 7.0.26 [33] using models selected via Modeltest and 1000 bootstrap replicates. MLSA and *cpn60* nucleotide sequences are available as supplementary data (S1 Dataset, S2 Dataset).

## Results

### *cpn60* accurately constructs species groupings

**Fig 1:**
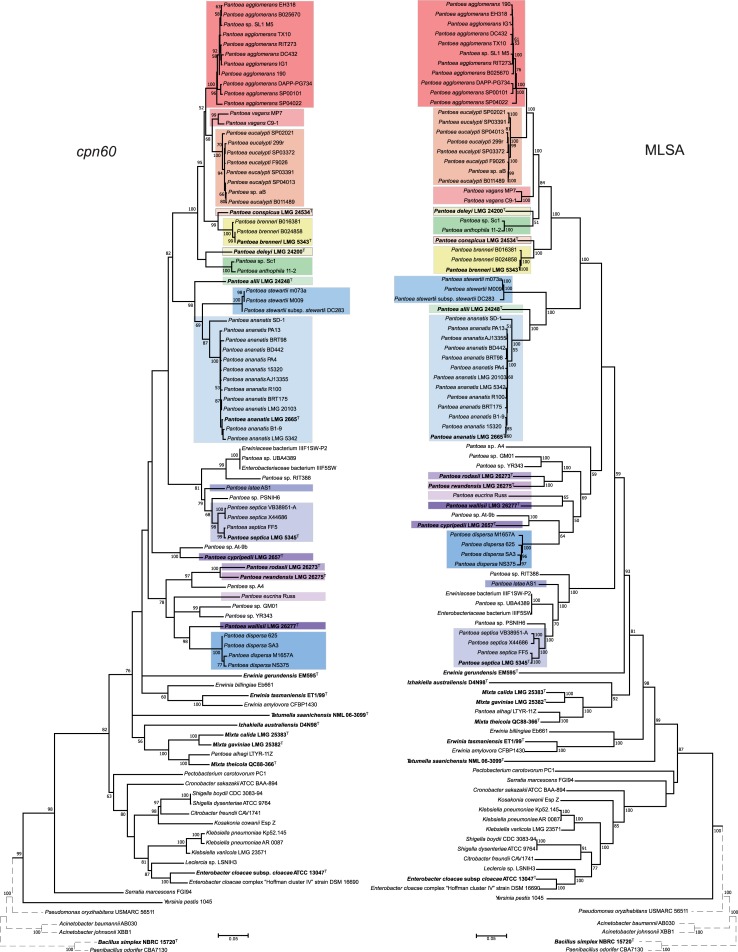
Phylogenies of reference *Pantoea* strains and related genera generated using the *cpn60* gene (left tree) and concatenated sequences of the *fusA*, *gyrB*, *leuS*, *recA*, *rpoB*, *rplB*, and *atpD* genes (right tree). Phylogenies were generated using the Maximum likelihood algorithm, general time reversible model, and with values at nodes representing bootstrap values from 1000 replicates. Only values ≥ 50% are shown. Colored boxes indicate known *Pantoea* species groups. Outgroups beyond *Enterobacteriales* are denoted by dotted-line branches and are not to scale. Type strains are denoted by bold font and superscript "T".

A *cpn60* phylogenetic tree was constructed and compared to a seven gene (*fusA*, *gyrB*, *leuS*, *recA*, *rpoB*, *rplB*, and *atpD*) MLSA phylogenetic tree from representative *Pantoea* genomes, along with representative genera of the *Enterobacteriales*. The majority of clades corresponding to individual *Pantoea* species groups were largely consistent between the two trees, and supported by strong bootstrap values, although the relative positions of some clades differed between the two trees (Fig 1). For example, the *P*. *agglomerans* group forms a sister group to *P*. *eucalypti* in the MLSA tree with *P*. *vagans* forming the basal group whereas in the *cpn60* tree *P*. *vagans* is a sister group to *P*. *eucalypti* with *P*. *agglomerans* forming the basal group (Fig 1). There were similar incongruencies noted for the positions of the majority of *Pantoea* clades (Fig 1); however, in all these cases, taxa of the same species always formed monophyletic groups, but their recent common ancestor with other species varied.

### One quarter of clinical strains labeled *Pantoea* are misidentified

**Fig 2:**
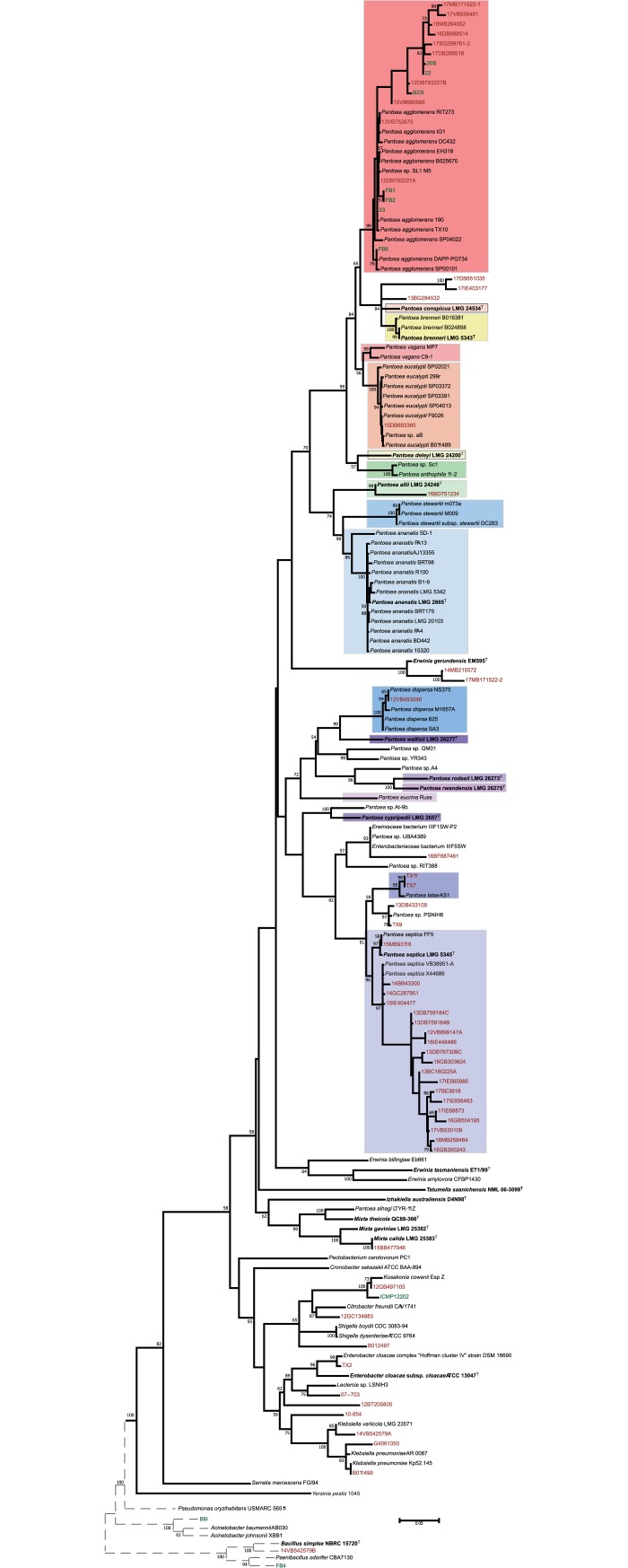
Phylogeny of clinical isolates and reference strains generated using *cpn60* sequences. The phylogeny was generated using maximum likelihood with the general time reversible model. Nodes represent the result of 1000 bootstrap replicates, with values ≥ 50% shown. Colored boxes indicate established *Pantoea* species groups. Outgroups beyond *Enterobacteriales* are denoted by dotted-line branches and are not to scale. Type strains are denoted by bold font and superscript "T". Clinical strains analyzed in this study are shown in red font while environmental strains are shown in green font.

The nucleotide sequence of the *cpn60* gene from 64 bacterial isolates that had been received as *Pantoea* were added to the previously established *cpn60* tree shown in Fig 1. Of these candidate *Pantoea* isolates, 54 were obtained from patients while 10 were collected from the environment (Table 1). Of the 54 clinical isolates, 47 were initially identified by MALDI-TOF, 4 were initially identified by a combination of VITEK 2 and 16S rRNA gene, 2 were initially identified via MicroScan combined with 16S rRNA gene and biochemical typing and a single isolate was initially identified using VITEK 2 ID cards. The 10 environmental isolates were initially identified visually by pigmentation. Based on the *cpn60* phylogeny, 47 of the 64 isolates were confirmed to belong to the genus *Pantoea* (Fig 2, Table 1). These included 17 *P*. *agglomerans*, 1 *P*. *allii*, 1 *P*. *dispersa*, 1 *P*. *eucalypti*, 19 *P*. *septica*, 2 strains of the proposed species *P*. *latae* [34], and 6 *Pantoea* sp. with 3 found in the *P*. *brenneri*/*P*. *conspicua* lineage and 3 found in the *P*. *septica*/*P*. *latae* lineage possibly representing new species (Fig 2). Of the 17 *P*. *agglomerans* strains, 10 were clinical and were associated with sepsis, wound infection, and esophageal tracheal combitube contamination, while the other seven were isolated from flea beetles and various plant sources (Table 1). All of the other strains in the other species groups of *Pantoea* were clinical in origin. *P*. *septica* strains, which accounted for almost half of all *Pantoea* clinical isolates identified in this study were associated with a variety of medical conditions, including renal failure, febrile neutropenia, leg ulcer infection, foot ulcer infection, and conjunctivitis (Table 1). Strains identified as the proposed species *P*. *latae* were obtained from blood and sputum while the single *P*. *dispersa* strain was obtained from a contaminated esophageal tracheal combitube in a patient who had suffered cardiac arrest (Table 1). Of the three *Pantoea* sp. falling in the *P*. *brenneri*/*P*. *conspicua* lineage, 13BG284532 was obtained from the tracheal secretions of a premature infant while 17DB651035 and 17IE403177 were associated with urinary tract infection and endotracheal secretions (Table 1). The partial *cpn60* sequence of 13BG284532 was more similar to the *cpn60* sequence of the *P*. *conspicua* type strain than any *P*. *brenneri* strain (S2 Table) and therefore it may belong to *P*. *conspicua*. 17DB651035 and 17IE403177 share 99% nucleotide identity with the *P*. *brenneri* type strain over a 770 bp region of their 16S rRNA gene, suggesting they likely belong to the *P*. *brenneri* species group. There was also some ambiguity in the identity of the other three *Pantoea* sp. strains, 13DB433109, 16BF887461, and TX9, which grouped with reference *Pantoea* strains that have not been assigned to any existing species. Strains 13DB433109 and TX9, isolated from urine and a wound on the foot respectively, both grouped with *Pantoea* sp. PSNIH6 as part of a sister taxon to *P*. *latae* (Table 1, Fig 2). Strain 16BF887461, isolated from the blood of a 1 year old patient, grouped with *Pantoea* sp. UBA4389 and *Pantoea* sp. RIT388, forming a sister taxon to the *P*. *septica*/*P*. *latae* lineage (Table 1, Fig 2). These may represent more divergent *P*. *latae* strains or new species. Finally, one strain obtained from the maxillary sinus of a patient with chronic sinusitis (Table 1), forms a sister taxon to *P*. *allii* (Fig 2), and shares 99% identity to the *P*. *allii* type strain in a 721 bp region of the 16S rRNA gene, indicating that this strain belongs to the *P*. *allii* species group.

Of the 17 isolates that were not *Pantoea*, two were environmental isolates that grouped with the Gram-negative *Acinetobacter* and the Gram-positive *Paenibacillus*, and one was a clinical isolate identified as *Bacillus* by *cpn60* (Fig 2, Table 1). The remaining 14 isolates included 13 clinical and 1 environmental isolate, all of which clustered within the *Enterobacteriales*. The identity of 9 of the 13 clinical strains was established by *cpn60* and 16S rRNA gene analysis, as well as phylogenetic grouping (S2 Table). Among these were representatives of *Citrobacter*, *Enterobacter*, *Erwinia*, *Klebsiella*, *Kosakonia* and *Mixta* (Table 1). The identification of clinical *Erwinia* strains was unexpected, and these grouped within a newly defined species group, *Erwinia gerundensis*, which in both our MLSA and *cpn60* trees was non-monophyletic with the other *Erwinia*. The 16S rRNA gene of our clinical *E*. *gerundensis* strains shared 100% identity with the *E*. *gerundensis* type strain 16S rRNA gene over approximately 800 bp. The identities of the other 4 of the 13 clinical strains and the lone remaining environmental isolate, ICMP12202, were ambiguous. Strains 10–854 and ICMP12202 matched different genera depending on whether *cpn60* or 16S rRNA gene was used. 10–854 is identified by the RDP database with confidence as *Pseudocitrobacter* (92%), while cpnDB initially returned *Leclercia* and *Klebsiella* as best hits until *Pseudocitrobacter* representatives were included (S2 Table). ICMP12202, a strain previously identified as *Kosakonia* [35], and which our *cpn60* analysis also confirms to be *Kosakonia* is identified as a weak *Citrobacter* (42%) by the RDP based on the 16S rRNA gene (S2 Table). *Kosakonia* is included in Training Set 16 of the RDP Classifier. Three strains, B012497, 12BT205805, and 07–703 also had conflicting 16S rRNA gene and *cpn60* matches, and were categorized as “*unclassified Enterobacteriaceae*” by the RDP database. The groupings of these strains in our *cpn60* phylogeny were ambiguous, and could not be used to assign identity with any confidence.

## Discussion

This study identified clinical and environmental candidate *Pantoea* isolates to the species-level using *cpn60*. Our phylogenetic analysis examining the evolutionary history of *cpn60* using representatives of each *Pantoea* species showed that these formed monophyletic groups consistent with those found in the MLSA trees, indicating largely vertical maintenance of the *cpn60* locus within species groups. Notably, the relative position of each clade in the *cpn60* tree differed from that of the MLSA tree (Fig 1), suggesting that at least for *Pantoea*, this locus may not accurately reconstruct the evolutionary history of the species groups. Similar results were obtained with previous studies evaluating the *leuS* gene, which has been suggested to have value as a single gene identification system for *Pantoea* isolates despite minor differences in species group relationships between MLSA phylogenies compared to *leuS* [22]. Other studies have also shown that *cpn60* effectively identifies clinical isolates of *Campylobacter* to the species-level, as well as the opportunistically invasive Actinobacterium, *Gardnerella vaginalis*, which was consistent with the taxonomic classification obtained by whole-genome-based methods [36]. The congruence of our MLSA and *cpn60* trees and the robust confidence values (Fig 1) indicates that *cpn60* provides adequate phylogenetic information to accurately assign a given *Pantoea* isolate to a species group, or in the case of ambiguously positioned sequences, assign it to a specific multi-species lineage.

An unexpected result was the identification of two clinical isolates of the recently proposed *E*. *gerundensis*, a species group whose type strain was isolated from the leaves of a pear tree [37]. The members of the genus *Erwinia* have not been documented as opportunistic human pathogens, and when coupled with the non-monophyly of *E*. *gerundensis* with the other *Erwinia* species in either tree (Fig 1), the placement of *E*. *gerundensis* within the genus *Erwinia* remains uncertain. In addition, the *cpn60* gene of *E*. *gerundensis* is more similar to that of members of *Pantoea* than it is to other members of *Erwinia*. This could account for why *E*. *gerundensis* appeared within the *Pantoea* lineage of the candidate isolate-containing *cpn60* phylogeny, albeit with low branch support (Fig 2). The reliability of *cpn60* for the identification of *Erwinia* strains should therefore be explored further.

Using *cpn60* we determined that of the 64 candidate *Pantoea* strains (54 clinical, 10 environmental), 47 were confirmed to be *Pantoea*. Of the 54 clinical strains, 41 were correctly identified, leaving one quarter misidentified. 81% (38/47) of clinical isolates initially identified via MALDI-TOF were correctly identified while 3 of 4 strains initially identified using VITEK combined with 16S rRNA gene typing were correctly identified. The remaining 3 clinical isolates, initially identified with other methodological combinations, were misidentified. Because these strains were classified prior to the description of the proposed *Mixta calida*, we considered the single *M*. *calida* strain found in our study to be correctly identified [38]. Three non-*Pantoea* strains, 07–703, 12BT205805, and B012497 are sufficiently divergent that they remain unclassified. Of the clinical strains that were confirmed to be *Pantoea*, the vast majority belonged to *P*. *agglomerans* (10 strains) and *P*. *septica* (19 strains). *P*. *agglomerans* has been previously identified as a human pathogen [6,39,40]; however, it has been suggested that *P*. *agglomerans* may be incorrectly considered a human pathogen due to inaccurate identification of clinical isolates and nomenclatural confusion [10]. Strains that were misidentified as *Pantoea* tended to be other closely related enteric species (S2 Table), many of which are genera that contain opportunistic, multi-drug resistant human pathogens [41,42]; however, many clinical strains identified were confirmed to be *P*. *agglomerans*. The recurrent isolation of strains implicated in sepsis, esophageal tracheal combitube contamination, and various wound infections suggests that *P*. *agglomerans* is not simply guilty by association (Table 1) [1]. In contrast, *P*. *septica* appears to be largely clinical in origin [1,43], so it was not surprising that 19 strains were recovered from patients suffering from a variety of conditions, including renal failure, respiratory failure, ulcers, infected wounds, and conjunctivitis (Table 1). Other species of *Pantoea* that were recovered included *P*. *dispersa* and strains within the *P*. *brenneri/P*. *conspicua* lineage, with all three aforementioned species having been noted by the scientific community to be human-associated species groups that have been isolated from the clinical environment previously [1,43–45]. For example, *P*. *dispersa* has been implicated as the cause of bacteremia and multiple cases of neonatal sepsis while *P*. *brenneri* and *P*. *conspicua* have been isolated from human sputum and blood respectively [43,46,47].

A single clinical isolate of *P*. *allii* was identified in our study, which to our knowledge is the first instance of this plant-pathogenic species being isolated from the clinical environment. *P*. *allii* has been characterized as a plant pathogen able to cause disease in onion and is most closely related to *P*. *ananatis* and *P*. *stewartii* [48]. As *P*. *allii* is closely related to *P*. *ananatis*, a species group that has also been isolated from the clinical environment and has been described as an opportunist [5,49,50], it is possible that *P*. *allii* also carries host-association and virulence factors that may enable opportunism. Similarly, *P*. *eucalypti* has been isolated predominantly from diseased plants [4] and is generally considered a plant-associated species, yet our study has identified one clinical isolate of *P*. *eucalypti*, marking the third clinical strain of *P*. *eucalypti* that we have reported [1]. While the isolation of *P*. *allii* from the clinical environment has been the exception rather than the rule, species like *P*. *eucalypti* are becoming more frequently identified among clinical specimens. Similarly, we identified two clinical strains of the proposed species, *P*. *latae*, the type strain of which had been isolated from the rhizosphere of cycad plants and forms a sister group to the *P*. *septica* lineage [34]. Both of these clinical strains fall, with confidence, within the *P*. *latae* species group (Fig 2). Although *P*. *latae* has previously only been isolated from plants, the identification of clinical isolates in our study is not necessarily surprising given that *P*. *septica* is so closely related to *P*. *latae* (Fig 1). It is possible that some of the factors responsible for the ability of *P*. *septica* to persist in the clinical environment are shared with *P*. *latae*. This could also explain the clinical origin of 13DB433109, 16BF887461, and TX9 which grouped with reference *Pantoea* strains related to the *P*. *septica*/*P*. *latae* lineage that have yet to be assigned to a species (Fig 2).

There is mounting evidence that clinical specimens of *Pantoea* are not simply misidentifications caused by incomplete MALDI-TOF spectral databases; rather, it is possible that the genetic factors used by *Pantoea* strains for environmental persistence and for association with plants, insects and other hosts are being co-opted and used for establishing opportunistic human infections [51,52]. For example, in the closely related clinically-isolated species *M*. *calida*, a plant type III secretion system was identified suggesting that some of these strains may have other primary hosts [53]. Although there is little information on the genetic determinants that may be used by *Pantoea* strains for opportunistic association with humans, some factors have been identified that may play a role in infection. Many strains secrete a diversity of natural products, some having antimicrobial activity against clinically relevant pathogens [54,55], while others are biosurfactants that exhibit cytotoxicity toward animal cells [56]. It has recently been reported that *P*. *septica* and the *P*. *ananatis*/*P*. *stewartii* lineage have horizontally acquired the biosynthetic gene cluster responsible for production of the iron-gathering siderophore, aerobactin, which is absent in other *Pantoea* species [57]. Aerobactin is a known virulence factor and has been demonstrated to be essential for the virulence of hypervirulent, *Klebsiella pneumoniae* [58,59]. While these genetic factors may have evolved to exploit very specific niches, they may provide a competitive edge in other environments as well, which may include the human host environment.

Although we were able to successfully identify the isolates in our study to the species level using *cpn60*, there are several limitations to single gene identification methods including limited phylogenetic signal, and misleading evolutionary histories due to horizontal transfer events. Using multiple gene or whole genome-based identification methods would likely yield more accurate and precise results; however, this requires additional time and resources with currently available technologies. These constraints, particularly in the clinical setting, are easily overcome by MALDI-TOF, since it is relatively inexpensive, fast and accurate. Still, our work has demonstrated that *Pantoea* isolates continue to be misidentified by MALDI-TOF, although this may continue to improve as MALDI-TOF spectra for *Pantoea* are expanded with additional representatives of the species groups and their close relatives. This also raises questions about the frequency of isolates that are *Pantoea*, but are being incorrectly identified as other genera. Our work has shown that *P*. *septica* and *P*. *agglomerans* continue to account for a large portion of clinical *Pantoea* isolations from urinary tract infections, wound infections, conjunctivitis, sepsis, renal failure, sinusitis, ulcers, and febrile neutropenia. Furthermore, our work shows that species of *Pantoea* considered primarily plant pathogens can be isolated from humans, although their specific involvement in disease establishment and pathology still requires further investigation.

## Supporting information

S1 Dataset Nucleotide sequences of concatenated MLSA genes in FASTA format. (TXT)

S2 Dataset Nucleotide sequences of *cpn60* genes in FASTA format. (TXT)

S1 Table Accession numbers of reference strains used for phylogenetic and cpnDB analyses. (XLSX)

S2 Table Strain identification based on *cpn60* and 16S rRNA typing. (XLSX)
